# A Nutrient Ratio-Based, Web-Enabled Food Quality Score Is Associated With Weight and Blood Pressure Compared With Leading Nutrient Profiling Systems

**DOI:** 10.1016/j.cdnut.2026.109383

**Published:** 2026-05-29

**Authors:** Christopher J Damman, Cara L Frankenfeld

**Affiliations:** 1 Department of Medicine, Division of Gastroenterology, University of Washington, Seattle, WA, United States; 2 GutBites MD, Seattle, WA, United States; 3 MaineHealth Institute for Research, Scarborough, ME, United States

**Keywords:** nutrient ratios, obesity, hypertension, nutrient profiling systems, diet quality

## Abstract

**Background:**

Rising prevalence of chronic cardiometabolic conditions may be partly driven by shifts in dietary patterns. Nutrient profiling systems (NPSs) aim to guide healthier food choices through labeling and consumer-facing technologies but vary in accessibility and how well they distinguish food healthfulness.

**Objectives:**

The primary objective was to compare a web-enabled, ratio-based NPS, Nutrient Consume Score (NCS), and its underlying nutrient ratios with 4 other leading NPSs, Nutri-Score, Health Star Rating, NOVA Classification, and Food Compass 2.0, by examining associations with obesity and blood pressure. Secondary objectives included assessing associations with cardiometabolic biomarkers and identifying food categories contributing most to each score.

**Methods:**

Cross-sectional study of NHANES 2015–2016 data from 9971 adults aged ≥20 y were analyzed. Dietary intake was assessed using a single 24-h dietary recall (day 1), and NPS scores were calculated. Multivariable regression models adjusted for sociodemographic and health factors examined associations with obesity, blood pressure, and cardiometabolic biomarkers. Compositional analysis evaluated food categories driving scores.

**Results:**

Higher NCS was associated with lower BMI [*β* = −0.64 kg/m^2^ per 10-unit increase; 95% confidence interval (CI): −0.86, −0.41; *P* < 0.0001], waist circumference (*β* = −1.63 cm; 95% CI: −2.23, −1.02; *P* < 0.0001), systolic blood pressure (*β* = −1.01 mm Hg; 95% CI: −1.67, −0.35; *P* < 0.0051), and diastolic blood pressure (*β* = −0.56 mm Hg; 95% CI: −0.90, −0.23; *P* < 0.0026), with effect sizes comparable with other NPSs. Calorie-to-weight, sodium-to-potassium, and saturated-to-unsaturated fat ratios were associated with weight outcomes, whereas sodium-to-potassium and carbohydrate-to-fiber ratios were associated with blood pressure outcomes.

**Conclusions:**

The NCS was associated with weight and blood pressure in this cross-sectional analysis, with effect estimates comparable in magnitude with other commonly used NPSs. These findings support the potential utility of a ratio-based, web-enabled NPS for assessing diet quality in relation to cardiometabolic risk factors.

## Introduction

The rising incidence and prevalence of obesity [1], metabolic syndrome [2], and other noncommunicable disease [3] in the United States and globally [4] has been linked to shifts in the types of foods available to consumers including increased ultraprocessed [5] and hyperpalatable foods [6] with resulting changes in dietary habits [7]. Complex, confusing [8], and conflicting [9] nutrition guidance makes it difficult for individuals to make informed, healthy choices on food purchases, preparation, and consumption [10].

Nutrient profiling systems (NPSs) aim to simplify nutrition complexity into easier-to-follow ratings that assess the health quality of foods and beverages. These systems can offer real-time guidance through package labeling [11], online websites [12], and smartphone technologies [13,14], helping individuals make healthier food choices. Additionally, they can support public health recommendations [15] and guide food companies in creating healthier products [16,17].

Although there are over 100 reported NPSs [15,18], a small subset has been rigorously evaluated for their association with health outcomes. The most studied NPSs with strongest links to improved dietary choices and better health are Nutri-Score (NS) [19], Health Star (HS) [20], NOVA Classification (NC) [21], and Food Compass (FC) [22,23].

NS [24], popular in several European countries, and HS [25], widely used in Australia and New Zealand, rank foods based on nutrient profiles and food categories. The NC categorizes foods by their degree of processing, popularizing the term ultraprocessed food [26]. FC evaluates foods across 9 health domains, including categories like nutrient ratios, fiber, and phytochemicals [23,27]. These systems are variably effective in assessing the relative quality of different ultraprocessed foods [28] and not available on front of product packaging in many countries including the United States [23].

Despite the availability of multiple NPSs, a gap remains for a rigorously studied, web-enabled algorithm that is independent of country-specific labeling practices and that can scale, based on relative rather than absolute nutrient measures. The Nutrient Consume Score (NCS) [12] is a recently developed web-based algorithm that focuses on nutrient ratios—carbohydrate-to-fiber [29], saturated fat-to-unsaturated fat [30], sodium-to-potassium [31], and calorie-to-weight [32]—as simple, measurable proxies for the degree of food processing and previously identified as strong predictors of food quality [33]. It also incorporates alcohol and adjusts for food categories high in bioactive components that support a healthy gut microbiome, including polyphenols, bioactive fats, fermentable fibers, and fermentation products [34].

The primary aim of this study was to compare NCS, a nutrient ratio-based, web-enabled food quality score with other leading NPSs in relation to weight and blood pressure, key prognostic factors in cardiometabolic disease and mortality. We hypothesized that NCS would demonstrate associations with these outcomes that are at least comparable in magnitude with those observed for other commonly used NPSs. Secondary aims included evaluating associations with cardiometabolic biomarkers and identifying food categories contributing most to each score.

## Methods

### Data sources and analytic population

The NHANES is a repeated, cross-sectional analysis that has been conducted continuously since 1999 and data are released in 2-y cycles, with exceptions for recent cycles because of COVID-19 disruptions in survey data collection [35]. The survey includes interview and physical measurement components and is conducted in a nationally representative sample of ∼5000 persons each year from persons located in 15 counties that are randomly selected each year.

For the primary analysis to compare NCS and other NPSs, data from the continuous survey data releases 2015–2016 were used for this analysis because complete previously published data were available for all evaluated NPS scores for this NHANES data cycle [36]. In the 2015–2016 data cycle, 9971 individuals participated. The following exclusions were applied in this order to obtain the analytic subsample used (*n* excluded): age <20 y (*n* = 4252), missing education (*n* = 5), missing poverty-to-income ratio (PIR) (*n* = 0), missing day 1 dietary intake data (*n* = 699), physiologically unsustainable low dietary intake of <500 kcal (*n* = 42), physiologically unsustainable high dietary intake >5000 kcal (*n* = 57), missing smoking information (*n* = 20), missing waist circumference (*n* = 203), missing BMI (*n* = 9), missing blood pressure (*n* = 51), or missing physical activity (*n* = 35). NHANES does not have missing data for age, gender, or race/ethnicity. The analytic sample for primary analyses used included 4598 adult individuals. A secondary analysis was undertaken to evaluate associations of the NCS score with other cardiometabolic biomarkers in NHANES cycles 2005–2018. Additional exclusions to the 4598 individuals in the analytic sample were missing LDL, HDL, or total cholesterol, plasma glucose, or blood triglycerides (*n* = 2647). The analytic sample for secondary analyses of cardiometabolic biomarkers included 1951 adult individuals.

### Dietary intake assessment

Dietary intake is assessed for NHANES using 2 multiple pass 24-h recalls. The first 24-h recall is conducted in-person during the visit to the Mobile Examination Center (MEC). The second 24-h recall is conducted via phone 3 to 10 d after the MEC visit. All NHANES participants are eligible for the 24-h recall interviews. The interviews are conducted using a midnight-to-midnight time frame for the 24-h period before the interview. The USDA Food Survey Research Group conducts the dietary data collection methodology, maintenance of databases used to code and process data, and data review and processing. The dietary data are released in 2 files: individual foods and total nutrients. The individual foods file lists each food reported by the participant, along with details of the consumption such as a USDA Food and Nutrient Database for Dietary Studies (FNDDS) code (food code), eating occasion, and amount of food/beverage consumed in grams. The total nutrient intake file is a daily aggregate of nutrients from the reported foods consumed for that 24-h recall.

### NCS and nutrient ratio scores

NCS is an online NPS based on nutrient ratios as proxies for the degree of food processing—carbohydrate-to-fiber, saturated fat-to-unsaturated fat, sodium-to-potassium, and calorie-to-weight—along with additional nutrients (protein, alcohol, vitamin D, iron, calcium) available on United States Nutrition Facts labels. All scores used in this paper are available through the online portal (https://gutbites.org/carb-fiber-ratio-calculator).

Positive adjustments are made for food categories like fruits, vegetables, nuts, seeds, and whole grains to account for microbiome-supportive and bioactive factors not listed on food labels: polyphenols, prebiotic fibers, bioactive fats, and fermentation products. In addition, negative adjustments are made for alcohol, soft drinks, processed meats, and processed potatoes to reflect additives and food components linked to negative health outcomes. Three versions of NCS were evaluated in the analysis: NCS proper, NCS without alcohol included in calculations to compare with other algorithms that do not include alcohol, and NCS without bioactive adjustments.

Nutrient ratios: carbohydrate-to-fiber (fiber ratio), saturated fat-to-unsaturated fat (fat ratio), sodium-to-potassium (salt ratio), and calorie-to-weight (energy ratio) were also analyzed separately to evaluate their independent contributions to observed associations. Nutrient ratio scores were mapped to a quantized scale of 1 to 3 in 0.125 increments.

### Other NPSs

To contextualize the performance of the NCS, we compared it with several leading and widely used NPSs that represent distinct approaches to assessing food quality.

NS is a scoring algorithm that balances positively weighted nutrients (e.g., fruits, vegetables, and fiber) against negatively weighted ones (e.g., saturated fat, sugars, and sodium), resulting in a color-coded rating from A to E [24].

HS Rating separates foods into 3 groups—dairy products, nondairy beverages, and all other foods—and assigns points based on levels of protein, fats, saturated fats, energy, carbohydrates, sugar, and sodium, yielding in a 5-star scale with half-star increments [25].

The NC ranks foods by processing level, distinguishing between minimally or unprocessed foods, processed ingredients, processed foods, and ultraprocessed foods, resulting in a 4-level scale [26].

FC assesses foods across 9 domains—nutrient ratios, vitamins, minerals, food-based ingredients, additives, processing, specific lipids, fiber and protein, phytochemicals—producing a 100-point score [23,27].

### Score calculation and weighting

NCS values were retrieved using the NCS algorithm available online at Gutbites.org [12]. Each of the 4 other NPSs was rescaled to enable direct comparison with NCS. NS (A–E = 100–20), HS Rating (0.5–5 = 10–100), NC (1–4 = 80–20), and FC 2 (1–100 = 1–100) scores were derived from previously reported values for foods within the 2015–2016 What We Eat in America (WWEIA)/FNDDS food categories [23,27,37]. Alcoholic beverages were assigned null values in systems that did not evaluate them: NS, HS, NC, and FC.

All scores and nutrient ratios were evaluated in 3 ways: weighted by calorie (kcal), weighted by weight (grams), and unweighted. Unweighted scores were calculated for each individual as the mean score of each food consumed in the 24-h period divided by the total number of food items. Scores weighted by energy and weight were calculated as the sum of the individual food items’ scores multiplied by the kcal or grams provided by the food item and then divided by the total kcal or grams for the 24-h period. cores were analyzed as continuous variables on a 10-unit scale.

### Food categories and contributions to scores

To evaluate which foods were contributing to most of the variation in the NPS and ratio scores, a compositional approach was applied. Individual foods reported were assigned to food group classifications based on the WWEIA food categories [38]. This analysis was performed for all NPSs. Total intake of food categories was calculated for each person based on percentage energy (kcals) provided by all the foods in that category for the 24-h dietary intake recall period. Center-log transformed ratios of percentage energy contributed by food categories to overall energy were calculated [39] using the R *compositions* package [40] to include in regression models.

### Outcomes

Anthropometry and blood pressure were evaluated as continuous outcomes and as binary outcomes based on established cutoffs. Anthropometric characteristics were measured during the in-person visit by trained technicians. Blood pressure was measured 1 to 4 times by trained technicians, and the mean of available measurements for each participant was used in analyses. Details about measurement and analysis are available from NHANES established procedures. The mean of the available measurements for each person was used for analysis.

Obesity was defined as BMI ≥30 kg/m^2^ [41] and abdominal obesity was defined as waist circumference >88 cm for females and >102 cm for males [42]. High blood pressure was defined as the presence of systolic blood pressure 130 mm Hg or diastolic blood pressure 85 mm Hg [42,43].

Blood-based cardiometabolic risk factors (analysis conducted for NCS only to maintain consistency across NHANES cycles where comparable data for other NPSs were not available) evaluated included LDL, HDL, total cholesterol, and triglycerides, and plasma glucose. These risk factors were evaluated as continuous measures and in relation to established cutoffs for high or low values [42,43]. Additionally, metabolic syndrome was assessed and was classified as the presence of ≥3 of: abdominal obesity, high blood pressure, high LDL, low HDL, or elevated serum glucose [42].

### Covariates

Personal demographic characteristics were included to describe the analytic group and as adjustment variables in multivariable analyses. Characteristics included were age (in categories for description and in years for multivariable analyses), gender (female and male), race or ethnicity (non-Hispanic White, non-Hispanic Black, non-Hispanic other, and Hispanic), education (<high school graduate; high school graduate or equivalent; some college or associate degree; and, 4-y college graduate or more), PIR (<1, 1 to <2, 2, to <3, and 3+), smoking status (<100 cigarettes in lifetime; former smoker; current <20 cigarettes/d; and, current 20+ cigarettes/d), and physical activity [<15, 15 to <75, 75 to <165, 165+ metabolic equivalent task score (METS) per week]. Physical activity was assessed using self-reported leisure-time activity data and converted to MET-min/wk using standard NHANES algorithms, based on activity frequency, duration, and intensity.

### Statistical analysis

For visualization of overall differences in relation to increasing NCS score, descriptive statistics for covariates were calculated by tertiles of the energy-weighted NCS score. Means and SEs were calculated for continuous variables and frequencies and percentages were calculated for categorical variables. To evaluate associations of each NPS and nutrient ratio score with anthropometry, blood pressure, and cardiometabolic biomarkers, multivariable linear and logistic regression analyses were performed for continuous and binary outcomes, respectively, and adjusted for demographic characteristics (age, gender, race/ethnicity, education, PIR, smoking, and exercise). For food category contributions to scores, linear regression analyses were used to evaluate the contribution of center-log ratio transformed food categories of the energy-weighted scores and subscores. Except for the compositional analysis of food category contributions to NPSs and nutrient ratios scores, analyses were conducted using population weights supplied by NHANES and incorporating the complex survey design. A subpopulation command was used to maintain appropriate population weights for participants with complete data included in the analysis. Statistical significance was set at *P* < 0.05 for analyses with cardiometabolic and mortality outcomes. Statistical analysis was conducted using Stata (StataCorp, version 18).

## Results

### Study population characteristics

Study population characteristics by tertiles of energy-weighted NCS scores are presented (Table 1). Distributions of demographic and behavioral characteristics varied across tertiles; however, formal statistical comparisons between groups were not performed, and results should be interpreted descriptively. Categories more represented in the highest score tertiles included age >65, female gender, Hispanic, non-Hispanic other, 4-y college graduates, PIR of ≥3, and never smokers. Categories more represented in the lowest score tertiles included younger age, non-Hispanic White, non-Hispanic Black, lower education level, current smoker, and highest physical activity level (165+ METS).

**TABLE 1:** Study population characteristics by tertiles of energy-weighted NCS score in NHANES adults, 2015–2016

Personal characteristics	Tertile 1 (<37.08), *n* = 1533	Tertile 2 (37.08–48.31), *n* = 1533	Tertile 3 (>48.31), *n* = 1532
Age (y)			
20–34	458 (33.3%)	370 (25.4%)	360 (25.4%)
35–49	415 (26.8%)	392 (24.8%)	339 (22.6%)
50–64	380 (25.2%)	434 (28.6%)	403 (27.4%)
65+	280 (14.8%)	337 (21.2%)	430 (24.7%)
Gender			
Male	764 (53.3%)	752 (47.1%)	693 (43.0%)
Female	769 (46.7%)	781 (52.9%)	839 (57.0%)
Race and non-Hispanic ethnicity			
Non-Hispanic White	589 (67.7%)	559 (68.0%)	420 (58.2%)
Non-Hispanic Black	415 (13.8%)	316 (10.4%)	220 (7.6%)
Non-Hispanic Other	116 (4.9%)	199 (8.2%)	333 (16.2%)
Hispanic	413 (13.6%)	459 (13.4%)	559 (18.0%)
Education			
<HS graduate or equivalent	293 (12.4%)	324 (12.8%)	394 (13.5%)
HS graduate or equivalent	412 (26.9%)	347 (21.6%)	264 (13.8%)
Some college or associates degree	527 (36.1%)	463 (33.4%)	389 (30.6%)
Four-year college graduate or more	301 (24.6%)	399 (32.2%)	485 (42.2%)
Poverty-to-income ratio			
<1	307 (14.7%)	265 (10.2%)	312 (13.5%)
1 to <2	399 (19.4%)	387 (20.5%)	338 (15.0%)
2 to <3	252 (18.0%)	262 (16.9%)	219 (12.8%)
≥3	460 (41.6%)	488 (45.8%)	500 (49.8%)
Missing	115 (6.3%)	131 (6.6%)	163 (8.9%)
Smoking			
Never	807 (51.6%)	869 (55.4%)	1009 (64.1%)
Former	338 (23.7%)	360 (26.7%)	365 (25.4%)
Current, <20 cig/d	282 (16.0%)	242 (14.2%)	139 (8.6%)
Current, 20+ cig/d	106 (8.6%)	62 (3.7%)	19 (2.0%)
Physical activity (METS/wk)			
<15	666 (38.2%)	680 (40.4%)	688 (36.1%)
15 to <75	431 (31.3%)	453 (32.7%)	510 (41.2%)
75 to <165	219 (15.8%)	195 (14.4%)	197 (13.6%)
165+	217 (14.7%)	205 (12.5%)	137 (9.0%)

Abbreviations: METS, metabolic equivalent task score; NCS, Nutrient Consume Score.

### Weight and blood pressure associations with NPS scores

**FIGURE 1:**
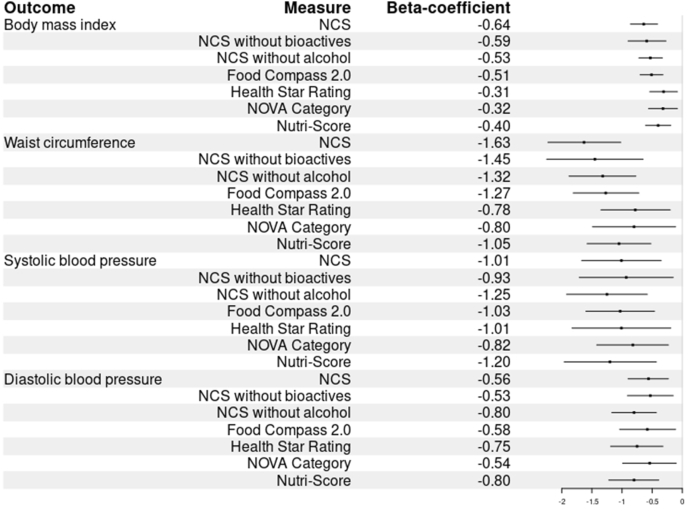
Associations of nutrient profile scores (in 10-unit values) with BMI, waist circumference, and blood pressure. Beta-coefficients from multivariable-adjusted models are displayed. Models are adjusted for age, gender, race or Hispanic ethnicity, education, smoking, poverty-income ratio, and physical activity.

All energy-weighted NPS scores were associated with continuous measures of anthropometry in multivariable-adjusted models (Figure 1; Supplemental Table 1). For NCS, higher scores were associated with lower BMI [*β* = −0.64 kg/m^2^ per 10-unit increase; 95% confidence interval (CI): −0.86, −0.41; *P* < 0.0001] and waist circumference (*β* = −1.63 cm; 95% CI: −2.23, −1.02; *P* < 0.0001). Associations with blood pressure were also consistent across indices. For NCS, higher scores were associated with lower systolic blood pressure (*β* = −1.01 mm Hg; 95% CI: −1.67, −0.35; *P* < 0.0051) and diastolic blood pressure (*β* = −0.56 mm Hg; 95% CI: −0.90, −0.23; *P* < 0.0026).

**FIGURE 2:**
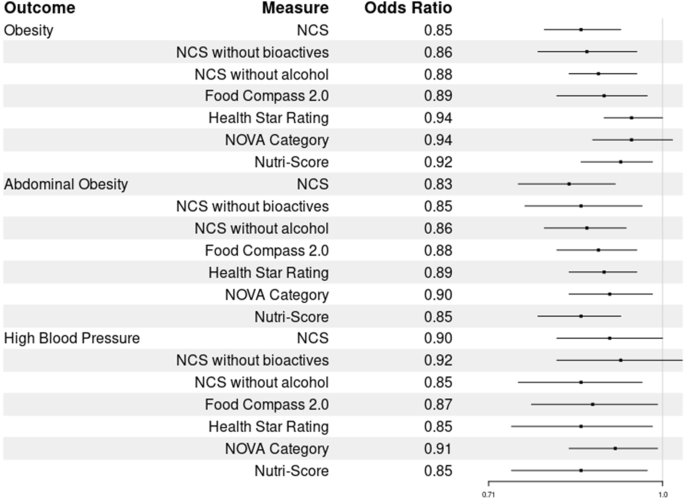
Associations of nutrient profile scores (in 10-unit values) with obesity, abdominal obesity, and high blood pressure. Odds ratios from multivariable-adjusted models are displayed. Models are adjusted for age, gender, race or Hispanic ethnicity, education, smoking, poverty-income ratio, and physical activity.

Higher energy-weighted scores for NCS, FC, and NS were associated with lower odds of obesity, whereas all NPSs were associated with lower odds of abdominal obesity (Figure 2; Supplemental Table 1). For NCS, higher scores were associated with lower odds of obesity [odds ratio (OR) = 0.85; 95% CI: 0.75, 0.91; *P* < 0.0004] and abdominal obesity (OR = 0.83; 95% CI: 0.75, 0.91; *P* < 0.0007). All NPSs except NCS (OR = 0.90; 95% CI: 0.81, 1.00; *P* < 0.0586) were associated with blood pressure although effect sizes were smaller.

Modifications to NCS influenced associations. Removing bioactive adjustments attenuated associations with anthropometric outcomes and, in some cases, rendered associations with high blood pressure nonsignificant. In contrast, excluding alcohol increased the significance of associations with high blood pressure while attenuating associations with obesity.

### Weight and blood pressure associations with nutrient ratio scores

**FIGURE 3:**
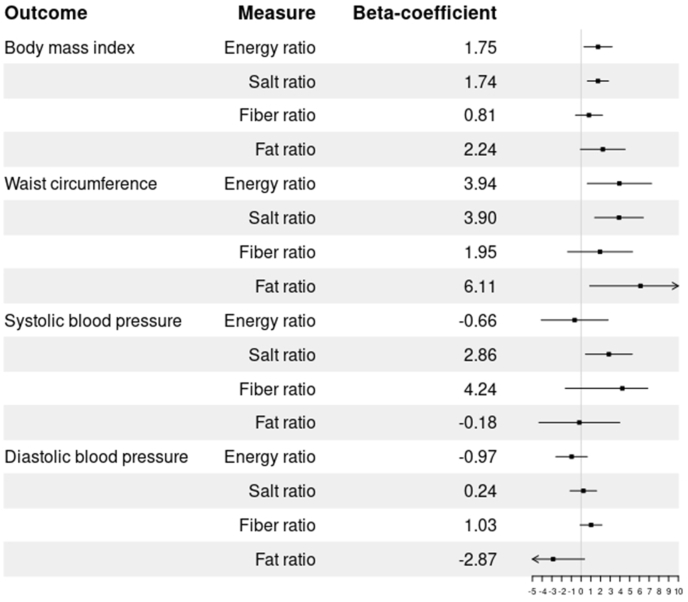
Associations of nutrient ratio scores (in 1-unit values) with BMI, waist circumference, and blood pressure. Beta-coefficients from multivariable-adjusted models are displayed. Models are adjusted for age, gender, race or Hispanic ethnicity, education, smoking, poverty-income ratio, and physical activity. Fat ratio = saturated-to-unsaturated fat ratio; Salt ratio = sodium-to-potassium ratio; Fiber ratio = carbohydrate-to-fiber ratio; Energy ratio = calorie-to-weight ratio.

**FIGURE 4:**
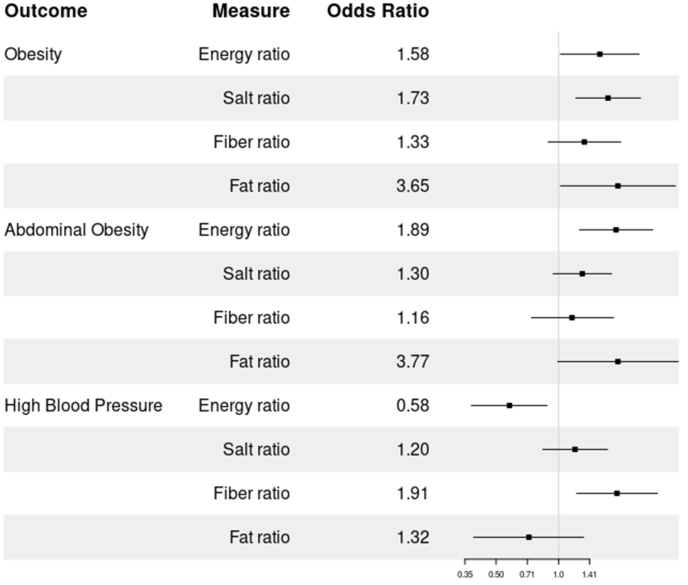
Associations of nutrient ratio scores (in 1-unit values) with obesity, abdominal obesity, and high blood pressure. Odds ratios from multivariable-adjusted models are displayed. Models are adjusted for age, gender, race or Hispanic ethnicity, education, smoking, poverty-income ratio, and physical activity. Fat ratio = saturated-to-unsaturated fat ratio; Salt ratio = sodium-to-potassium ratio; Fiber ratio = carbohydrate-to-fiber ratio; Energy ratio = calorie-to-weight ratio.

Nutrient ratio scores (carbohydrate-to-fiber, saturated-to-unsaturated fat, sodium-to-potassium, and calorie-to-weight) were analyzed separately (Figures 3 and 4), with higher energy-weighted values reflecting less favorable nutrient profiles.

Higher energy, salt and fat ratio scores were variably associated with higher weight-related measures, with the energy ratio most consistently associated across outcomes. In contrast, the fiber ratio showed no association with weight measures.

Associations with blood pressure varied by ratio. For systolic and diastolic blood pressure, only the salt ratio showed a consistent positive association. For high blood pressure, the fiber ratio was positively associated, whereas the energy ratio was inversely associated.

### Other cardiometabolic risk factor associations with NPS and nutrition ratio scores

Among cardiometabolic biomarkers (Supplemental Figures 2A, B and 3A, B), higher NCS (all versions) and FC scores were associated with higher total cholesterol. Only higher NCS scores without bioactives were associated with higher triglycerides. Higher carb ratios were associated with lower HDL cholesterol, whereas higher salt ratios were associated with higher plasma glucose.

### Food category contributors to NPS scores

**FIGURE 5:**
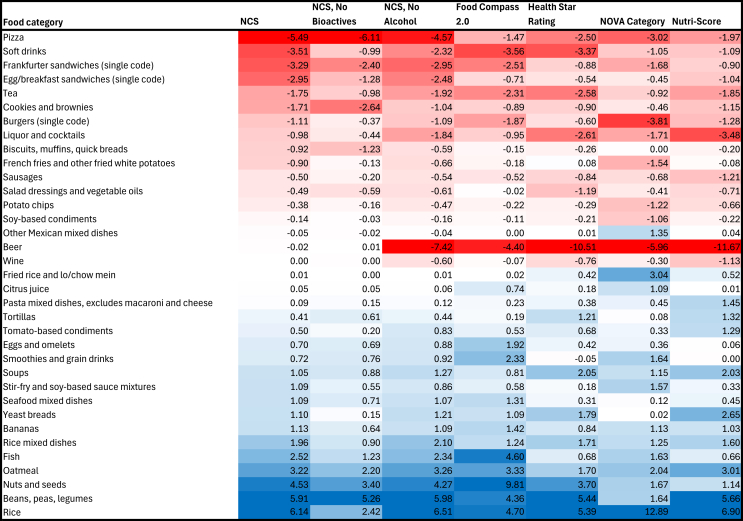
Heatmap of food category contributions to kcal-weighted scores. Values represented are the percentage of variation explained (*R*^2^∗100∗indicator for direction of association). Food categories that had no consumption in the study population and foods not included in a WWEIA category are not included, and only foods for which at least it explains ≥1% of variation of a score is included. Darker shading represents more variation explained, and red indicates inverse associations and blue indicates positive associations. WWEIA, What We Eat in America.

Food category contributions to NPS variation were broadly consistent across scoring systems (Figure 5; Supplemental Figure 4). Categories contributing most to lower scores included pizza, soft drinks, frankfurters, and egg/breakfast sandwiches, whereas categories contributing most to higher scores included rice, beans and legumes, and nuts and seeds. The relative contribution of specific categories varied modestly across scoring systems.

## Discussion

NPSs assess the quality of food and beverages, and aim to help consumers, industry, and government select, create, and promote healthier options to improve public and individual health [18]. This study shows that leading NPSs are associated with weight and blood pressure, key prognostic factors in cardiometabolic disease, neurological disorders, cancer, and mortality. A ratio-based web-available score performed similarly to leading NPSs and also associated with a higher cholesterol, likely driven by high favorable HDL. Notably, the food categories contributing the most to scores were largely consistent across systems. Those foods contributing most to positive scores included minimally processed foods and those that contributed most to negative scores included ultraprocessed foods.

A key finding is that much of the power of NPSs may be captured in the following nutrient ratios: carbohydrate-to fiber [29], saturated fat-to-unsaturated fat [30], sodium-to-potassium [31], and calorie-to-weight [32], which all associated with weight and variably associated with blood pressure. Nutrient ratios reflect nutritional balance in whole foods and the degree to which certain nutrients are concentrated or depleted in processed foods. Ratios are explicitly incorporated in systems like NCS and FC and implicitly applied in systems like NS and HS, which assign positive and negative weights to underrepresented and overrepresented nutrients. Nutrient ratios may complement the NC, which has been critiqued for grouping all ultraprocessed foods together, by offering an empirical approach to assessing the relative healthfulness of different ultraprocessed foods [44]. The unfavorable association of calorie-to-weight with blood pressure could be related to beverages (including juices and sodas) having lower nutrient density and future work may benefit from treating nutrient density of beverages and foods differently.

Another key finding is that including alcohol in a ratio-based score may enhance its health-discriminatory power for weight, but not blood pressure, perhaps also owing to complexities around beverages. In NPSs that excluded alcohol from their calculations, alcoholic beverages contributed to the scores, likely due to co-consumption of alcohol with other foods. This result also highlights an important point about the role of alcohol in overall dietary intake for individuals who choose to consume alcohol. Alcohol contributes ≥16% of the mean United States adult’s daily caloric intake [45], a figure that is likely higher since the impact of COVID-19 on consumption [46]. However, many are unaware of alcohol’s impact [47]. Given the prevalence of alcohol consumption and its association with obesity [48] and other adverse health outcomes [49], there is a need for consumer education and NPSs that account for alcohol’s caloric contribution to encourage healthier dietary choices.

A third key takeaway is that removing adjustments for food groups serving as proxies for bioactive factors diminished the ability of NCS to discriminate associations with weight and blood pressure measures. The observed association between NCS without bioactives and higher triglycerides may reflect the loss of triglyceride-lowering effects typically contributed by bioactive components. These food group adjustments included both those linked to positively associated microbiome-active and bioactive factors (e.g., phytochemicals, fermentable fibers, and bioactive fats) and those linked to negatively associated bioactives (e.g., alcohol, fructose, nitrites, acrylamide, advanced glycosylation end products, and trans fats). A better understanding and measurement of these positive and negative health-associated bioactive factors, along with their explicit inclusion in food labels and NPS algorithms, are key focuses of current nutrition research [34].

NPSs have been shown to positively influence consumer buying habits [50] and prospective cohort studies have shown that they can positively impact health measures like obesity and mortality [19,51]. They can play a key role in public health messaging through front-of-package labels [11]. In countries where front-of-package labels do not yet exist, web-enabled portals [12], and smartphone technologies that scan product Universal Product Codes (UPCs) to determine food quality could be useful [13,14]. A ratio-based score in particular could intuitively encourage individuals to incorporate more fiber-, potassium-, unsaturated fat-rich foods, potentially countering the health impact of foods high in simple carbohydrates, sodium, and saturated fat. A ratio-based NPS could also guide the food industry in developing healthier products by providing empirical guide rails to rebalance simple carbohydrates with fiber [52,53] and sodium with potassium [54].

NPSs hold promise for improving health outcomes, but they have limitations. Some systems generate outlier results for certain foods that conflict with epidemiological data [55]. Most exclude alcohol, despite its strong association with adverse health outcomes [19,27,56], whereas others are critiqued for being overly simple or too complex [22]. Additionally, most NPSs rely on nutritional databases that lack quantification of the bioactive components of foods, such as microbiome-active components (i.e., polyphenols, fermentable fibers, and fermentation products), and are missing data on food additives that may harm the microbiome and individual [57]. Most NPSs fail to account for a food’s matrix or ultrastructure, which may be important for understanding the health benefits of unprocessed foods [58]. NPS algorithms do not currently provide personalized advice that may be important to account for individual variations in nutrient requirements, including those influenced by the gut microbiome [59,60]. Tailoring algorithms in low- and middle-income country cohorts, where baseline macronutrient and micronutrient intakes differ significantly, may also be necessary.

This study design also has limitations including selection [61], recall [62], and reporting [63] biases. Although NHANES attempts to survey respondents to obtain representativeness to the United States population, there is still self-selection of participants who are invited to participate in NHANES, and individuals may misreport information in surveys and interviews because of misremembering or aligning responses with social desirability. Additionally, the single assessment of dietary intake may not be reflective of longer term or usual intake. However, the large sample size of NHANES does mitigate some of these biases. Temporality of associations cannot be determined because of the cross-sectional design of NHANES, and weight measures in particular can be subject to reverse causation bias [64]. Results regarding weight should be interpreted in this context, and it is possible that individuals of different body sizes eat differently rather than the scores predict weight. Cohort studies demonstrating prospective association between NS and abdominal obesity suggest that reverse causation may not entirely explain the associations observed [19]. In addition, this analysis focuses on criterion validity by evaluating cross-sectional associations with selected cardiometabolic outcomes. Other aspects of validity, including predictive, discriminant, and divergent validity, as well as reliability of the NCS, have not yet been fully established and warrant further study in longitudinal and interventional settings.

In conclusion, this study highlights the potential of NPSs including a nutrient ratio-based web-enabled score to support personal and public health efforts in curbing weight gain and improving blood pressure. Ratio-based systems, in particular, may guide individuals, food companies, and governments in rebalancing nutrients in our diet. As a ratio-based system, NCS may be particularly useful at measuring the spectrum of health of different processed and ultraprocessed foods (Supplemental Figure 1). A ratio-based system also has advantages in being independent of absolute values of nutrients so it operates independent of scale and allows assessing food combinations. Although NPSs offer valuable insights, most do not account for alcohol and are primarily supported by correlative research. NCS also offers an advantage over other algorithms given its web accessibility. Future studies leveraging smartphone access to the web-enabled algorithm could assess NCS’s capacity for personalization, generalizability across diverse populations, and causal effects on metabolic health.

## Author contributions

The authors’ responsibilities were as follows – CJD: designed and conducted the research, drafted the paper, and was responsible for final content; CLF: performed the statistical analysis, wrote the methods section, and revised the manuscript; both authors: have read and approved the final version.

## Data availability

NHANES data used in this work are freely available from the National Center for Health Statistics. Other data described in the manuscript and a code book will be made available upon request pending approval.

## Declaration of Generative AI and AI-assisted Technologies in the Writing Process

During the preparation of this manuscript, ChatGPT was used on a limited basis to improve clarity and wording of the text. The authors subsequently reviewed and revised all content. No generative AI tools were used for data analysis or interpretation.

## Funding

The authors reported no funding received for this study.

## Conflicts of interest

CLF is an Associate Editor for Journal of Nutrition and Annals of Epidemiology and on the Editorial Board for Critical Reviews in Food Science and Nutrition, and works as a consultant for EpidStrategies, A BlueRidge Life Sciences Company. CJD is editor-in-chief at GutBites MD, a not-for-profit website aimed at making gut health research accessible. He is on the scientific advisory board at Supergut, One Bio, and Oobli, and has equity ownership in these companies.

## Figures and Tables

**Figure:**
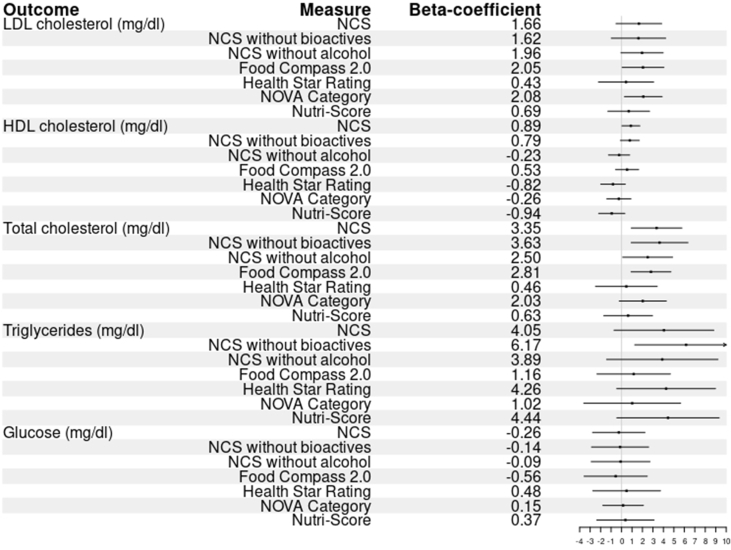
Supplemental Figure 1. Distribution of NCS scores for representative foods, organized by food category.

**Figure:**
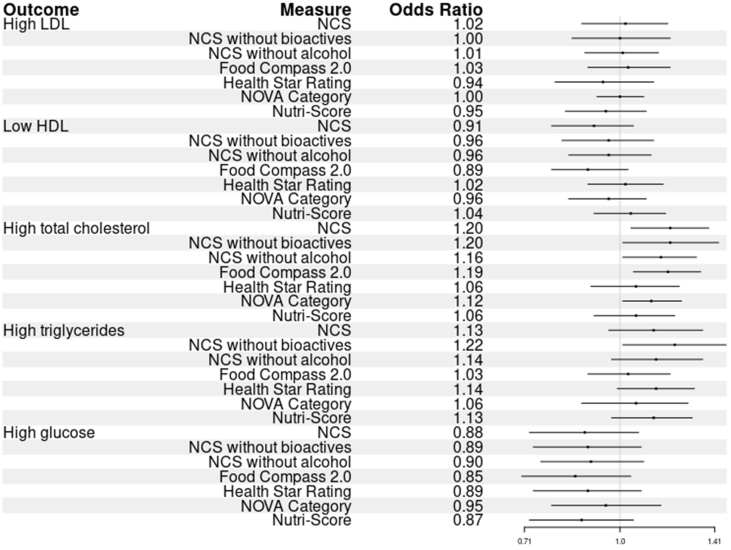
Supplemental Figure 2. Associations of nutrient profile scores (in 10-unit values) with high LDL cholesterol, low HDL cholesterol, high total cholesterol, high triglycerides, and high plasma glucose. Beta-coefficients for continuous outcomes (panel A) and odds ratios for dichotomous outcomes (panel B) from multivariable adjusted models are displayed. Models adjusted for age, gender, race or Hispanic ethnicity, education, smoking, poverty-income ratio, and physical activity.

**Figure:**
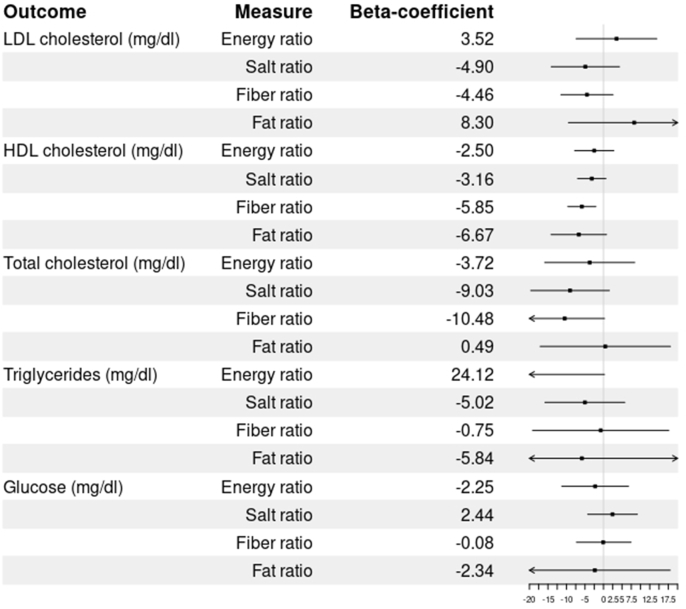
Supplemental Figure 3. Associations of nutrient ratios (in 1-unit values) with LDL cholesterol, HDL cholesterol, total cholesterol, triglycerides, and plasma glucose. Beta-coefficients for continuous outcomes (panel A) and odds ratios for dichotomous outcomes (panel B) from multivariable adjusted models are displayed. Models are adjusted for age, gender, race or Hispanic ethnicity, education, smoking, poverty-income ratio, and physical activity.

**Figure:**
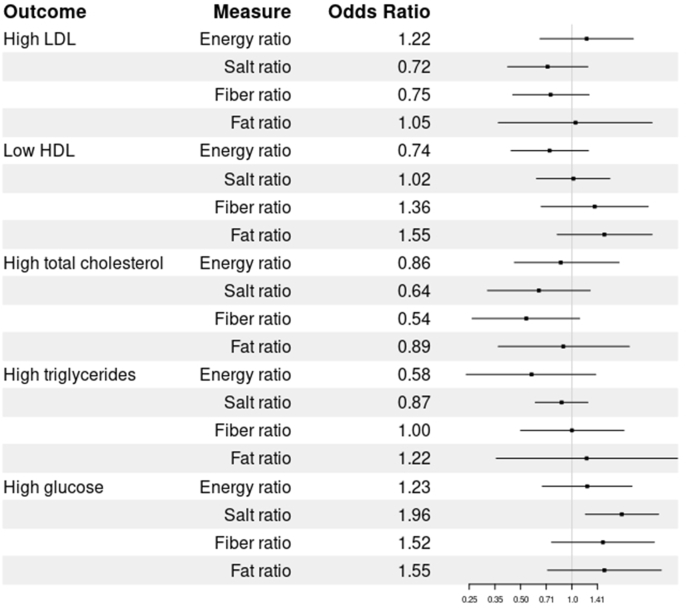
Supplemental Figure 4. Heatmap of food category contributions to kcal-weighted scores. Values represented are the percentage of variation explained (R-squared∗100∗indicator for direction of association). All WWEIA food categories displayed. Darker shading represents more variation explained, and red indicates inverse associations and blue indicates positive associations.
