# Supplementary material for: A Nutrient Ratio-Based, Web-Enabled Food Quality Score Is Associated With Weight and Blood Pressure Compared With Leading Nutrient Profiling Systems
Source: Curr Dev Nutr. 2026 May 29;10(7):109383. doi: 10.1016/j.cdnut.2026.109383 (PMC13355711; doi:10.1016/j.cdnut.2026.109383)
Supplement: Multimedia component 2 [file mmc2.pdf]

Food category	NCS	NCS, No Bioactives	NCS, No Alcohol	Food Compass 2.0	Health Star Rating	NOVA Category	Nutri-Score
Pizza	-5.49	-6.11	-4.57	-1.47	-2.50	-3.02	-1.97
Soft drinks	-3.51	-0.99	-2.32	-3.56	-3.37	-1.05	-1.09
Frankfurter sandwiches (single code)	-3.29	-2.40	-2.95	-2.51	-0.88	-1.68	-0.90
Egg/breakfast sandwiches (single code)	-2.95	-1.28	-2.48	-0.71	-0.54	-0.45	-1.04
Tea	-1.75	-0.98	-1.92	-2.31	-2.58	-0.92	-1.85
Cookies and brownies	-1.71	-2.64	-1.04	-0.89	-0.90	-0.46	-1.15
Burgers (single code)	-1.11	-0.37	-1.09	-1.87	-0.60	-3.81	-1.28
Liquor and cocktails	-0.98	-0.44	-1.84	-0.95	-2.61	-1.71	-3.48
Biscuits, muffins, quick breads	-0.92	-1.23	-0.59	-0.15	-0.26	0.00	-0.20
French fries and other fried white potatoes	-0.90	-0.13	-0.66	-0.18	0.08	-1.54	-0.08
Popcorn	-0.79	-0.98	-0.59	-0.10	-0.56	-0.34	-0.59
Cakes and pies	-0.59	-0.76	-0.44	-0.96	-0.57	-0.03	-0.76
Doughnuts, sweet rolls, pastries	-0.54	-0.85	-0.24	-0.60	-0.13	-0.10	-0.06
Tortilla, corn, other chips	-0.53	-0.90	-0.31	0.00	0.00	-0.11	-0.02
Sausages	-0.50	-0.20	-0.54	-0.52	-0.84	-0.68	-1.21
Salad dressings and vegetable oils	-0.49	-0.59	-0.61	-0.02	-1.19	-0.41	-0.71
Chicken/turkey sandwiches (single code)	-0.45	-0.39	-0.30	-0.35	0.00	-1.00	-0.01
Potato chips	-0.38	-0.16	-0.47	-0.22	-0.29	-1.22	-0.66
Cold cuts and cured meats	-0.32	-0.25	-0.48	-0.38	-0.53	-0.56	-0.37
Rolls and buns	-0.32	-0.91	-0.21	-0.74	-0.03	-0.71	0.00
Pretzels/snack mix	-0.31	-0.61	-0.25	-0.03	-0.06	-0.12	-0.02
Dark green vegetables, excludes lettuce	-0.30	-0.11	-0.40	-0.88	-0.15	-0.27	-0.25
Chicken patties, nuggets and tenders	-0.27	-0.21	-0.19	-0.13	-0.04	-0.46	-0.34
Candy containing chocolate	-0.23	-0.23	-0.11	-0.16	-0.58	-0.38	-0.24
Crackers, excludes saltines	-0.20	-0.22	-0.12	0.02	0.00	-0.04	-0.04
Pancakes, waffles, French toast	-0.19	-0.20	-0.10	-0.03	0.03	-0.02	0.02
Burritos and tacos	-0.19	-0.08	-0.06	-0.06	0.04	0.63	-0.04
Candy not containing chocolate	-0.19	-0.21	-0.29	-0.13	-0.17	-0.28	-0.24
Sport and energy drinks	-0.16	-0.23	-0.26	-0.40	-0.88	-0.04	-0.23
Spinach	-0.14	-0.11	-0.14	-0.11	-0.04	-0.12	-0.09
Soy-based condiments	-0.14	-0.03	-0.16	-0.11	-0.21	-1.06	-0.22
Mashed potatoes and white potato mixtures	-0.13	0.01	-0.16	0.01	0.01	-0.01	-0.35
Dried fruits	-0.11	-0.10	-0.12	-0.03	-0.10	-0.01	-0.18
Coffee	-0.11	-0.08	-0.04	-0.07	-0.15	0.00	-0.02
Baby food: fruit	-0.10	-0.04	-0.10	-0.05	-0.05	-0.26	-0.13
Cream cheese, sour cream, whipped cream	-0.09	-0.10	-0.13	-0.11	-0.33	-0.10	-0.17
Not included in a food category	-0.07	-0.07	-0.04	-0.05	0.00	-0.06	0.00
Tomatoes	-0.07	0.00	-0.07	-0.34	0.00	-0.20	-0.01
Peaches and nectarines	-0.06	-0.07	-0.08	-0.19	-0.07	-0.06	-0.04
Flavored milk, whole	-0.06	-0.06	-0.04	-0.26	-0.18	-0.08	-0.11
Sugars and honey	-0.05	-0.07	-0.10	-0.03	-0.29	-0.32	-0.20
Other Mexican mixed dishes	-0.05	-0.02	-0.04	0.00	0.01	1.35	0.04
Flavored milk, reduced fat	-0.05	-0.11	-0.03	-0.15	-0.05	-0.34	-0.01
Berries	-0.03	0.00	-0.05	-0.24	0.00	-0.07	-0.05
Beef, excludes ground	-0.03	-0.01	-0.03	-0.27	-0.09	0.47	-0.60
Dips, gravies, other sauces	-0.03	-0.06	-0.02	0.00	-0.02	-0.19	-0.03
Margarine	-0.02	-0.01	-0.02	-0.01	-0.02	0.01	0.00
Turnovers and other grain-based items	-0.02	-0.03	-0.01	0.01	0.02	0.26	-0.01
Shellfish	-0.02	-0.07	0.00	0.13	-0.06	0.08	-0.03
Apple juice	-0.02	-0.04	0.00	0.07	0.00	0.17	0.00
Corn	-0.02	-0.04	0.00	0.00	0.04	-0.07	0.04
Beer	-0.02	0.01	-7.42	-4.40	-10.51	-5.96	-11.67
Bacon	-0.01	-0.02	-0.02	0.02	-0.02	-0.03	-0.01
Citrus fruits	-0.01	-0.04	-0.02	-0.05	-0.08	0.00	-0.10
Fruit drinks	-0.01	-0.04	0.00	-0.12	-0.13	-0.08	-0.03
Frankfurters	-0.01	0.00	0.00	-0.17	-0.06	-0.11	-0.05
Cereal bars	-0.01	-0.05	0.00	0.05	-0.01	-0.02	0.00
Enhanced or fortified water	-0.01	-0.04	-0.01	-0.05	-0.03	-0.01	0.00
Cheese sandwiches (single code)	-0.01	-0.14	0.00	0.00	-0.20	-0.05	-0.14
Other vegetables and combinations	0.00	0.01	0.00	0.00	0.20	-0.02	0.16
Carrots	0.00	0.01	-0.01	-0.07	0.04	-0.05	0.02
Nachos	0.00	0.00	-0.01	-0.04	-0.05	-0.08	-0.02
Milk, nonfat	0.00	0.00	0.00	-0.01	0.01	0.00	0.03
Vegetable dishes	0.00	-0.03	0.00	0.03	-0.01	0.01	-0.01
Mustard and other condiments	0.00	0.00	0.00	-0.10	0.05	-0.08	0.02
Broccoli	0.00	-0.01	0.02	0.06	0.09	0.01	0.17
Bagels and English muffins	0.00	-0.22	0.00	-0.17	0.04	-0.32	0.10
Jams, syrups, toppings	0.00	0.00	0.00	0.02	0.00	0.00	-0.02
Grits and other cooked cereals	0.00	-0.01	0.00	-0.06	0.00	0.00	-0.02
Cabbage	0.00	0.00	0.00	-0.05	0.00	0.00	0.00
Seafood sandwiches (single code)	0.00	0.00	0.00	0.00	0.00	0.00	0.00

Blueberries and other berries	0.00	0.00	0.00	0.00	0.00	0.00	0.00
Pears	0.00	0.00	0.00	0.00	0.00	0.00	0.00
Pineapple	0.00	0.00	0.00	0.00	0.00	0.00	0.00
Vegetables on a sandwich	0.00	0.00	0.00	0.00	0.00	0.00	0.00
Tap water	0.00	0.00	0.00	0.00	0.00	0.00	0.00
Bottled water	0.00	0.00	0.00	0.00	0.00	0.00	0.00
Baby food: vegetable	0.00	0.00	0.00	0.00	0.00	0.00	0.00
Baby food: meat and dinners	0.00	0.00	0.00	0.00	0.00	0.00	0.00
Baby food: yogurt	0.00	0.00	0.00	0.00	0.00	0.00	0.00
Baby food: snacks and sweets	0.00	0.00	0.00	0.00	0.00	0.00	0.00
Baby juice	0.00	0.00	0.00	0.00	0.00	0.00	0.00
Baby water	0.00	0.00	0.00	0.00	0.00	0.00	0.00
Formula, ready-to-feed	0.00	0.00	0.00	0.00	0.00	0.00	0.00
Formula, prepared from powder	0.00	0.00	0.00	0.00	0.00	0.00	0.00
Formula, prepared from concentrate	0.00	0.00	0.00	0.00	0.00	0.00	0.00
Human milk	0.00	0.00	0.00	0.00	0.00	0.00	0.00
Saltine crackers	0.00	-0.01	0.00	0.00	0.01	0.00	0.04
Gelatins, ices, sorbets	0.00	0.00	0.03	0.01	0.00	0.00	0.00
Sugar substitutes	0.00	0.00	-0.01	-0.02	-0.09	0.25	-0.03
Nutrition bars	0.00	0.00	0.00	0.04	-0.04	-0.03	-0.03
Cheese	0.00	-0.10	0.01	0.07	-0.36	-0.02	-0.08
Liver and organ meats	0.00	0.02	0.00	0.07	0.00	0.08	0.00
Diet soft drinks	0.00	0.00	0.00	-0.02	-0.13	0.28	0.00
Other diet drinks	0.00	0.03	0.00	0.00	-0.01	-0.07	-0.01
Wine	0.00	0.00	-0.60	-0.07	-0.76	-0.30	-1.13
Fried vegetables	0.00	0.00	0.01	0.01	0.00	-0.01	0.00
Fried rice and lo/chow mein	0.01	0.00	0.01	0.02	0.42	3.04	0.52
String beans	0.01	0.01	0.00	-0.05	-0.01	-0.04	0.00
Protein and nutritional powders	0.01	0.00	0.01	0.06	-0.04	-0.01	-0.06
Other red and orange vegetables	0.01	0.00	0.00	0.00	0.00	0.01	0.00
Other sandwiches (single code)	0.01	0.00	0.02	-0.16	0.02	-0.57	0.07
Other dark green vegetables	0.01	0.03	0.00	0.00	0.00	0.10	0.03
Milk substitutes	0.01	0.03	0.00	-0.04	-0.01	0.00	0.01
Other fruit juice	0.01	0.02	0.01	0.39	0.01	0.45	0.00
Bean, pea, legume dishes	0.02	0.01	0.00	0.00	0.03	-0.01	0.03
Egg rolls, dumplings, sushi	0.02	-0.03	0.01	0.00	-0.01	0.25	0.00
Ready-to-eat cereal, higher sugar (>21.2g/100g)	0.02	0.00	0.02	0.44	0.09	0.02	0.04
Diet sport and energy drinks	0.02	0.02	0.01	0.00	0.01	0.00	0.02
Vegetable juice	0.02	0.08	0.03	0.11	0.00	0.00	0.00
Other fruits and fruit salads	0.02	0.02	0.00	0.01	0.02	0.00	0.03
Butter and animal fats	0.03	0.01	0.09	0.11	0.05	0.07	0.02
White potatoes, baked or boiled	0.03	0.00	0.03	0.10	0.03	0.33	0.00
Flavored milk, nonfat	0.04	0.04	0.13	0.03	0.22	0.01	0.37
Processed soy products	0.05	0.01	0.04	0.07	0.02	-0.07	0.05
Peanut butter and jelly sandwiches (single code)	0.05	0.00	0.10	0.05	0.01	-0.19	0.00
Flavored or carbonated water	0.05	0.09	0.02	-0.02	0.01	-0.02	0.02
Citrus juice	0.05	0.05	0.06	0.74	0.18	1.09	0.01
Turkey, duck, other poultry	0.05	0.10	0.06	0.19	0.27	0.11	0.07
Baby food: cereals	0.06	0.08	0.06	0.02	0.12	-0.01	0.14
Melons	0.06	0.03	0.02	0.29	0.00	0.08	0.01
Coleslaw, non-lettuce salads	0.06	0.04	0.02	0.04	0.03	0.05	0.03
Flavored milk, lowfat	0.06	0.07	0.07	-0.06	0.05	-0.17	0.05
Lamb, goat, game	0.06	0.04	0.09	0.14	0.04	0.32	0.05
Macaroni and cheese	0.07	0.14	0.07	0.01	0.03	0.02	-0.01
Milk shakes and other dairy drinks	0.07	0.17	0.13	-0.03	-0.02	-0.14	0.14
Mango and papaya	0.07	0.04	0.06	0.15	0.06	0.08	0.11
Nutritional beverages	0.09	0.12	0.08	0.11	-0.13	0.00	-0.02
Pasta, noodles, cooked grains	0.09	0.02	0.03	0.20	0.03	0.70	0.15
Pork	0.09	0.12	0.04	0.01	-0.05	0.21	-0.23
Pasta mixed dishes, excludes macaroni and cheese	0.09	0.15	0.12	0.23	0.38	0.45	1.45
Cream and cream substitutes	0.10	0.06	0.08	0.01	0.00	0.01	0.13
Grapes	0.12	0.06	0.08	0.10	0.06	0.01	0.13
Ice cream and frozen dairy desserts	0.13	0.05	0.29	-0.36	-0.03	-0.35	-0.07
Lettuce and lettuce salads	0.14	0.49	0.20	-0.01	0.97	0.11	0.45
Ground beef	0.15	0.14	0.13	0.04	-0.03	0.19	-0.07
Milk, lowfat	0.17	0.16	0.16	0.10	0.22	0.02	0.33
Apples	0.17	0.17	0.11	0.13	0.05	0.00	0.02
Onions	0.17	0.23	0.14	-0.01	0.07	0.07	0.09
Chicken, whole pieces	0.18	0.32	0.06	0.19	0.00	0.05	-0.23
Pasta sauces, tomato-based	0.20	0.34	0.14	0.23	0.13	0.06	0.01
Mayonnaise	0.20	0.24	0.16	0.32	0.05	0.32	0.03
Poultry mixed dishes	0.24	0.38	0.31	0.17	0.28	0.04	0.41
Ready-to-eat cereal, lower sugar (=<21.2g/100g)	0.24	0.28	0.30	0.58	0.57	-0.09	0.57

Vegetable mixed dishes	0.24	0.26	0.22	0.36	0.19	0.16	0.04
Cottage/ricotta cheese	0.25	0.20	0.22	0.10	0.13	0.00	0.06
Meat mixed dishes	0.26	0.33	0.33	0.01	0.83	0.42	0.63
Milk, reduced fat	0.28	0.20	0.54	0.51	0.68	0.98	0.66
Yogurt, regular	0.30	0.23	0.28	0.03	0.41	0.00	0.09
Yogurt, whole and reduced fat	0.35	0.36	0.34	0.49	0.19	0.19	0.16
Pudding	0.36	0.25	0.38	0.11	0.17	0.31	0.09
Tortillas	0.41	0.61	0.44	0.19	1.21	0.08	1.32
Yogurt, lowfat and nonfat	0.45	0.30	0.49	0.14	0.47	-0.05	0.38
Other starchy vegetables	0.46	0.08	0.39	0.64	0.46	0.54	0.20
Milk, whole	0.49	0.30	0.60	0.76	0.21	0.93	0.44
Tomato-based condiments	0.50	0.20	0.83	0.53	0.68	0.33	1.29
Olives, pickles, pickled vegetables	0.59	0.67	0.52	0.51	0.56	0.34	0.61
Eggs and omelets	0.70	0.69	0.88	1.92	0.42	0.36	0.06
Smoothies and grain drinks	0.72	0.76	0.92	2.33	-0.05	1.64	0.00
Yogurt, Greek	0.92	0.73	0.87	0.77	0.72	0.00	0.61
Soups	1.05	0.88	1.27	0.81	2.05	1.15	2.03
Stir-fry and soy-based sauce mixtures	1.09	0.55	0.86	0.58	0.18	1.57	0.33
Seafood mixed dishes	1.09	0.71	1.07	1.31	0.31	0.12	0.45
Yeast breads	1.10	0.15	1.21	1.09	1.79	0.02	2.65
Bananas	1.13	0.64	1.09	1.42	0.84	1.13	1.03
Rice mixed dishes	1.96	0.90	2.10	1.24	1.71	1.25	1.60
Fish	2.52	1.23	2.34	4.60	0.68	1.63	0.66
Oatmeal	3.22	2.20	3.26	3.33	1.70	2.04	3.01
Nuts and seeds	4.53	3.40	4.27	9.81	3.70	1.67	1.14
Beans, peas, legumes	5.91	5.26	5.98	4.36	5.44	1.64	5.66
Rice	6.14	2.42	6.51	4.70	5.39	12.89	6.90
