# Supplementary material for: A Nutrient Ratio-Based, Web-Enabled Food Quality Score Is Associated With Weight and Blood Pressure Compared With Leading Nutrient Profiling Systems
Source: Curr Dev Nutr. 2026 May 29;10(7):109383. doi: 10.1016/j.cdnut.2026.109383 (PMC13355711; doi:10.1016/j.cdnut.2026.109383)
Supplement: Multimedia component 3 [file mmc3.docx]

**Supplemental Table 1.** Associations of nutrient profile scores (in 10-unit values) with continuous measures of anthropometry and blood pressure, and with dichotomous measures for obesity, abdominal obesity, and high blood pressure. Results are derived from multivariable-adjusted models and presented using unweighted, gram-weighted, and kcal-weighted scoring approaches.

|  | **NCS** | **Food Compass 2.0** | **Health Star Rating** | **Nova Category** | **Nutri-Score** |
| --- | --- | --- | --- | --- | --- |
| **Multivariable regression results** | **beta/OR (CI), p-value** | **beta/OR (CI), p-value** | **beta/OR (CI), p-value** | **beta/OR (CI), p-value** | **beta/OR (CI), p-value** |
| **Unweighted** |  |  |  |  |  |
| Body Mass Index (kg/m2) | -0.86 (-1.18,-0.55), <0.0001 | -0.73 (-1.02,-0.43), 0.0001 | -0.72 (-1.05,-0.39), 0.0003 | -0.97 (-1.39,-0.55), 0.0002 | -0.78 (-1.13,-0.44), 0.0002 |
| Waist Circumference (cm) | -2.08 (-2.91,-1.26), 0.0001 | -1.73 (-2.48,-0.98), 0.0002 | -1.71 (-2.56,-0.87), 0.0006 | -2.32 (-3.41,-1.23), 0.0004 | -1.91 (-2.81,-1.01), 0.0004 |
| Systolic blood pressure (mmHg) | -0.65 (-1.28,-0.03), 0.0426 | -0.69 (-1.14,-0.24), 0.0055 | -0.73 (-1.53, 0.07), 0.0709 | -0.66 (-1.40, 0.07), 0.0729 | -0.84 (-1.66,-0.03), 0.0439 |
| Diastolic blood pressure (mmHg) | -0.30 (-0.65, 0.05), 0.0899 | -0.27 (-0.69, 0.15), 0.1915 | -0.52 (-0.86,-0.19), 0.0046 | -0.37 (-0.88, 0.13), 0.1382 | -0.49 (-0.89,-0.08), 0.0211 |
| Obese | 0.78 (0.71, 0.85), <0.0001 | 0.81 (0.73, 0.89), 0.0004 | 0.83 (0.75, 0.91), 0.0009 | 0.77 (0.68, 0.87), 0.0004 | 0.82 (0.74, 0.91), 0.0010 |
| Abdominal obesity | 0.81 (0.72, 0.92), 0.0034 | 0.83 (0.74, 0.93), 0.0032 | 0.82 (0.73, 0.92), 0.0023 | 0.76 (0.66, 0.87), 0.0009 | 0.81 (0.72, 0.91), 0.0015 |
| High blood pressure | 0.94 (0.86, 1.03), 0.1940 | 0.92 (0.84, 1.02), 0.1106 | 0.89 (0.80, 0.99), 0.0347 | 0.95 (0.85, 1.07), 0.4175 | 0.90 (0.80, 1.02), 0.0813 |
| **Weighted by grams** |  |  |  |  |  |
| Body Mass Index (kg/m2) | -1.00 (-1.29,-0.72), <0.0001 | -1.15 (-1.49,-0.80), <0.0001 | -0.86 (-1.14,-0.59), <0.0001 | -0.89 (-1.22,-0.57), <0.0001 | -0.70 (-0.93,-0.47), <0.0001 |
| Waist Circumference (cm) | -2.42 (-3.18,-1.66), <0.0001 | -2.69 (-3.58,-1.79), <0.0001 | -2.01 (-2.76,-1.26), <0.0001 | -2.10 (-2.94,-1.27), 0.0001 | -1.67 (-2.27,-1.07), <0.0001 |
| Systolic blood pressure (mmHg) | -0.04 (-0.63, 0.56), 0.9005 | -0.52 (-1.18, 0.15), 0.1190 | -0.39 (-1.01, 0.23), 0.1963 | -0.31 (-0.98, 0.36), 0.3419 | -0.33 (-0.90, 0.23), 0.2244 |
| Diastolic blood pressure (mmHg) | -0.05 (-0.35, 0.24), 0.7076 | -0.50 (-0.87,-0.12), 0.0124 | -0.41 (-0.69,-0.14), 0.0059 | -0.35 (-0.65,-0.06), 0.0233 | -0.26 (-0.50,-0.03), 0.0269 |
| Obese | 0.75 (0.69, 0.83), <0.0001 | 0.73 (0.64, 0.83), 0.0001 | 0.80 (0.73, 0.87), 0.0001 | 0.79 (0.70, 0.88), 0.0004 | 0.83 (0.78, 0.90), 0.0001 |
| Abdominal obesity | 0.76 (0.70, 0.84), <0.0001 | 0.74 (0.66, 0.83), <0.0001 | 0.79 (0.71, 0.88), 0.0003 | 0.79 (0.70, 0.89), 0.0009 | 0.82 (0.75, 0.91), 0.0008 |
| High blood pressure | 1.06 (0.98, 1.15), 0.1485 | 0.95 (0.85, 1.07), 0.4076 | 0.98 (0.90, 1.06), 0.5896 | 1.00 (0.92, 1.07), 0.8928 | 0.99 (0.92, 1.06), 0.7751 |
| **Weighted by kcal** |  |  |  |  |  |
| Body Mass Index (kg/m2) | -0.64 (-0.86,-0.41), <0.0001 | -0.51 (-0.70,-0.32), <0.0001 | -0.31 (-0.54,-0.08), 0.0124 | -0.32 (-0.56,-0.08), 0.0128 | -0.40 (-0.61,-0.19), 0.0012 |
| Waist Circumference (cm) | -1.63 (-2.23,-1.02), <0.0001 | -1.27 (-1.81,-0.72), 0.0002 | -0.78 (-1.35,-0.20), 0.0114 | -0.80 (-1.49,-0.11), 0.0253 | -1.05 (-1.58,-0.52), 0.0007 |
| Systolic blood pressure (mmHg) | -1.01 (-1.67,-0.35), 0.0051 | -1.03 (-1.60,-0.46), 0.0015 | -1.01 (-1.83,-0.19), 0.0187 | -0.82 (-1.42,-0.23), 0.0100 | -1.20 (-1.96,-0.43), 0.0045 |
| Diastolic blood pressure (mmHg) | -0.56 (-0.90,-0.23), 0.0026 | -0.58 (-1.04,-0.11), 0.0193 | -0.75 (-1.19,-0.32), 0.0022 | -0.54 (-0.99,-0.10), 0.0205 | -0.80 (-1.22,-0.39), 0.0009 |
| Obese | 0.85 (0.79, 0.92), 0.0004 | 0.89 (0.81, 0.97), 0.0138 | 0.94 (0.89, 1.00), 0.0359 | 0.94 (0.87, 1.02), 0.1117 | 0.92 (0.85, 0.98), 0.0207 |
| Abdominal obesity | 0.83 (0.75, 0.91), 0.0007 | 0.88 (0.81, 0.95), 0.0021 | 0.89 (0.83, 0.95), 0.0024 | 0.90 (0.83, 0.98), 0.0210 | 0.85 (0.78, 0.92), 0.0007 |
| High blood pressure | 0.90 (0.81, 1.00), 0.0586 | 0.87 (0.77, 0.99), 0.0399 | 0.85 (0.74, 0.98), 0.0297 | 0.91 (0.83, 0.99), 0.0333 | 0.85 (0.74, 0.97), 0.0235 |
